# AVPpred-BWR: antiviral peptides prediction via biological words representation

**DOI:** 10.1093/bioinformatics/btaf126

**Published:** 2025-03-27

**Authors:** Zhuoyu Wei, Yongqi Shen, Xiang Tang, Jian Wen, Youyi Song, Mingqiang Wei, Jing Cheng, Xiaolei Zhu

**Affiliations:** School of Information and Artificial Intelligence, Anhui Agricultural University, Hefei, Anhui 230036, China; School of Information and Artificial Intelligence, Anhui Agricultural University, Hefei, Anhui 230036, China; School of Information and Artificial Intelligence, Anhui Agricultural University, Hefei, Anhui 230036, China; School of Information and Artificial Intelligence, Anhui Agricultural University, Hefei, Anhui 230036, China; School of Science, China Pharmaceutical University, Nanjing 210009, China; School of Computer Science and Technology, Nanjing University of Aeronautics and Astronautics, Nanjing 210016, China; School of Information and Artificial Intelligence, Anhui Agricultural University, Hefei, Anhui 230036, China; School of Information and Artificial Intelligence, Anhui Agricultural University, Hefei, Anhui 230036, China

## Abstract

**Motivation:**

Antiviral peptides (AVPs) are short chains of amino acids, showing great potential as antiviral drugs. The traditional wisdom (e.g. wet experiments) for identifying the AVPs is time-consuming and laborious, while cutting-edge computational methods are less accurate to predict them.

**Results:**

In this article, we propose an AVPs prediction model via biological words representation, dubbed AVPpred-BWR. Based on the fact that the secondary structures of AVPs mainly consist of α-helix and loop, we explore the biological words of 1mer (corresponding to loops) and 4mer (4 continuous residues, corresponding to α-helix). That is, the peptides sequences are decomposed into biological words, and then the concealed sequential information is represented by training the Word2Vec models. Moreover, in order to extract multi-scale features, we leverage a CNN-Transformer framework to process the embeddings of 1mer and 4mer generated by Word2Vec models. To the best of our knowledge, this is the first time to realize the word segmentation of protein primary structure sequences based on the regularity of protein secondary structure. AVPpred-BWR illustrates clear improvements over its competitors on the independent test set (e.g. improvements of 4.6% and 11.0% for AUROC and MCC, respectively, compared to UniDL4BioPep).

**Availability and implementation:**

AVPpred-BWR is publicly available at: https://github.com/zyweizm/AVPpred-BWR or https://zenodo.org/records/14880447 (doi: 10.5281/zenodo.14880447).

## 1 Introduction

In recent years, efforts have intensified in combating various viral threats. As per World Health Statistics 2023 released by the World Health Organization (WHO), up to the 11th of March 2023, there had been over 759 million confirmed cases of COVID-19 and nearly 6.9 million reported COVID-19 deaths globally (2023). Existing antiviral drugs are limited and may have insufficient response, heightened resistance rates, and are associated adverse side effects (Duffy *et al.* 2008, Agarwal and Gabrani 2021).

Antiviral peptide (AVP) is a kind of antimicrobial peptide (AMP) that has the ability to fight against virus infection. Compared to commonly used antiviral drugs, AVPs not only demonstrate efficacy against re-emerging resistant viruses (Mahendran *et al.* 2020) but also possess natural, biodegradable properties with low toxicity. Most importantly, they can exhibit broad-spectrum activity against various viruses (Vilas Boas *et al.* 2019, Wang *et al.* 2022). Therefore, AVPs have recently attracted significant interest as an alternative to traditional chemical antiviral drugs.

AVPs can be obtained by traditional methods, including natural and biological sources (Zakaryan *et al.* 2021, Lima *et al.* 2022). However, traditional methods suffer from low yield and heavily rely on advanced instruments and skilled personnel, hindering their widespread application. On the other hand, the post-genomic era has witnessed a rapid increase in peptide sequences archived in various databases. Therefore, there is an urgent need to develop computational tools to efficiently discover novel AVPs.

In recent years, machine learning techniques have proven particularly powerful in handling large amounts of biomedical data and have been successfully applied for bioactive peptides prediction (Wei *et al.* 2017, Chowdhury *et al.* 2020, Li *et al.* 2020, Charoenkwan *et al.* 2021, Lv *et al.* 2022), which also have been utilized to facilitate detection of AVPs. AVPpred was the first tool built by support vector machines (SVMs) for AVPs prediction (Thakur *et al.* 2012). Chang and Yang pioneered the use of the random forest (RF) algorithm to predict AVPs (Chang and Yang 2013). ClassAMP was the first tool to predict peptides with antibacterial, antifungal, and antiviral activity using a multiple classification approach (Joseph *et al.* 2012). AI4AVP converted sequence data into matrices using PC6 coding and uses a Generative Adversarial Network (GAN) to increase the number of positive examples and balance the dataset (Lin *et al.* 2022). Deepstacked-AVPs utilizes a fused vector constructed from PSSM-TS (Tri-segmentation-based Position-Specific Scoring Matrix), word2vec-derived semantic features, and CTDT (Composition/Transition/Distribution-Transition) descriptors to address the limitations inherent in single encoding methods (Akbar *et al.* 2024). FEOpti-ACVP is pre-trained using the UniRep and BERT feature extraction frameworks, followed by an evaluation of various machine learning algorithms (Jiang *et al.* 2024).

Most previous machine learning-based approaches rely on hand-crafted features. Recently, there has been a shift toward using protein language models (pLMs) for predicting AVPs. For instance, Du *et al.* (2023) developed the UniDL4BioPep model based on the ESM-2 model. Similarly, Lobo *et al.* (2023) created a model based on the ESM1b and ProtT5. Despite their promising performance, these methods usually ignore the secondary structures of peptides which may provide additional useful information. Chowdhury *et al.* addressed this gap by leveraging physicochemical and structural properties of peptide sequences. Their feature selection analysis demonstrated that the secondary structures emerge as the most crucial feature (Chowdhury *et al.* 2020).

To efficiently extract the secondary structural information from peptide sequences, we proposed a concept of biological words used for pLMs. Specifically, considering that the secondary structures of small peptides mainly consist of α-helixes and loops, we utilized 1mer and 4mer of the peptide sequences as words to pre-train protein language models. Subsequently, the embeddings are extracted from these models for predicting AVPs. Experimental results verified that our approach effectively captures relevant sequence information and achieves superior prediction accuracy compared to existing models.

## 2 Materials and methods

### 2.1 Datasets

Pinacho-Castellanos *et al.* built comprehensive datasets from the largest experimentally validated nonredundant peptide dataset (Pinacho-Castellanos *et al.* 2021). In this study, we used the same datasets to build our model for fair comparison. For positive samples, the starPep tool was used to extract AVPs from starPepDB (Aguilera-Mendoza *et al.* 2019), and obtained 4653 AVPs. Then, those AVPs that have multiple activities such as antibacterial, antifungal, or antiparasitic activities were removed. For the remaining AVPs, only those with a length of 5–100 natural amino acids were kept. Thus, 2944 AVPs were obtained. For negative samples, a total of 561 046 peptide sequences without antimicrobial activity were downloaded from the UniProt (UniProt Consortium 2019) by retrieving the next two queries: (Golgi OR cytoplasm OR “endoplasmic reticulum” OR mitochondria) AND NOT antimicrobial AND length: [5 to 100], and NOT antimicrobial AND reviewed: YES. Then, the peptides containing non-natural residues were removed. For the remaining peptides, CD-HIT was used to filter sequences with >50% identity (Fu *et al.* 2012). At last, to balance the positive and negative samples, 2944 negative samples were selected. Finally, the dataset contains 2944 positive and 2944 negative samples, of which the training set accounts for 80% of the total dataset (4642 samples), the rest serves as the independent test dataset for model performance evaluation and comparison. Meanwhile, we used three other external test sets to further evaluate the generalization of our model. First, we collected updated AVPs in several databases such as DRAMP 4.0 (Ma *et al.* 2025), dbAMP 3.0 (Yao *et al.* 2025), APD3 (Wang *et al.* 2016), DBAASP v3 (Pirtskhalava *et al.* 2021), and so on, to build the first external test set. By the similar process mentioned above, we collected 3524 AVPs of which 2559 sequences are common with the sequences mentioned above, so we removed those sequences. For the remaining AVPs, CD-HIT were used to remove the redundancy with a sequence similarity threshold of 90%, and then obtained 475 AVPs. For negative samples, we collected the negative samples used in AI4AVP (Lin *et al.* 2022) and AVP-IFT (Guan *et al.* 2024). After removing duplicated samples with the training dataset, we randomly selected 475 negative samples. In addition, we used the dataset created by Xu *et al.* as the second external test set (Xu *et al.* 2024). Furthermore, we used an imbalance test set built by Pinacho-Castellanos *et al.* as the third external test set. Supplementary Table S1 shows the detailed information about the datasets.

As a binary classification experiment, we analyzed the distributions of sequence length of the positive and negative samples. Supplementary Fig. S1 indicates that the length distributions of positive and negative samples are similar, not biased.

### 2.2 Representation of biological words based on Word2Vec

Recognizing that most AVP peptides are mainly composed of α-helixes and loops, we chose to segment the peptide sequences into words with four residues and one residue, respectively. Because in the α-helix structure, the helix rises every 3.7 residues along the main axis. Thus, we suppose that extensive information can be extracted by using 4mer as a word in language models. In the field of natural language processing, multi-granularity text processing often facilitates the extraction of more information from sentences.

Given the biological words of the peptide sequences, Word2Vec is used to generate the embeddings of the words. Word2Vec includes two kinds of models: Continuous Bag of Words (CBOW) and Skip-gram (Sivakumar *et al.* 2020). In this study, CBOW is used to build Word2Vec models, which can extract sequence features for predicting target words based on context. Specifically, suppose the input of the CBOW is a sequence represented as $w_{1},\;w_{2},\;w_{3},\ldots ,\;w_{n}$, by biological words, a target words $w_{i}$, which can be predicted with the following objective function:


(1) $$Z=\frac{1}{N}\sum_{i=1}^{N}\log P(w_{i}|w_{i-t},\ldots ,w_{i-1},w_{i+1},\ldots ,w_{i+t}),$$

where $w_{i}$ represents the center word and $w_{i-t},\ldots ,w_{i-1},w_{i+1},\ldots ,w_{i+t}$ denote the context words. The conditional probability $P(w_{i}|w_{i-t},\ldots ,w_{i-1},w_{i+1},\ldots ,w_{i+t})$ is defined as follow:


(2) $$P\left(w_{i}|w_{i-t},\ldots ,w_{i-1},w_{i+1},\ldots ,w_{i+t}\right)=\frac{\exp(M_{i}^{T}h_{i})}{\sum_{h=1}^{j}\exp(M_{h}^{T}h_{i})}.$$

$M_{i}$ is column i of the weight matrix of the output layer. The $h_{i}$ is an N dimensional vector in hidden layer, which is computed by below equation.


(3) $$h_{i}=\frac{1}{C}V\times (\sum_{i=1}^{C}x_{i}).$$

**Figure 1.**
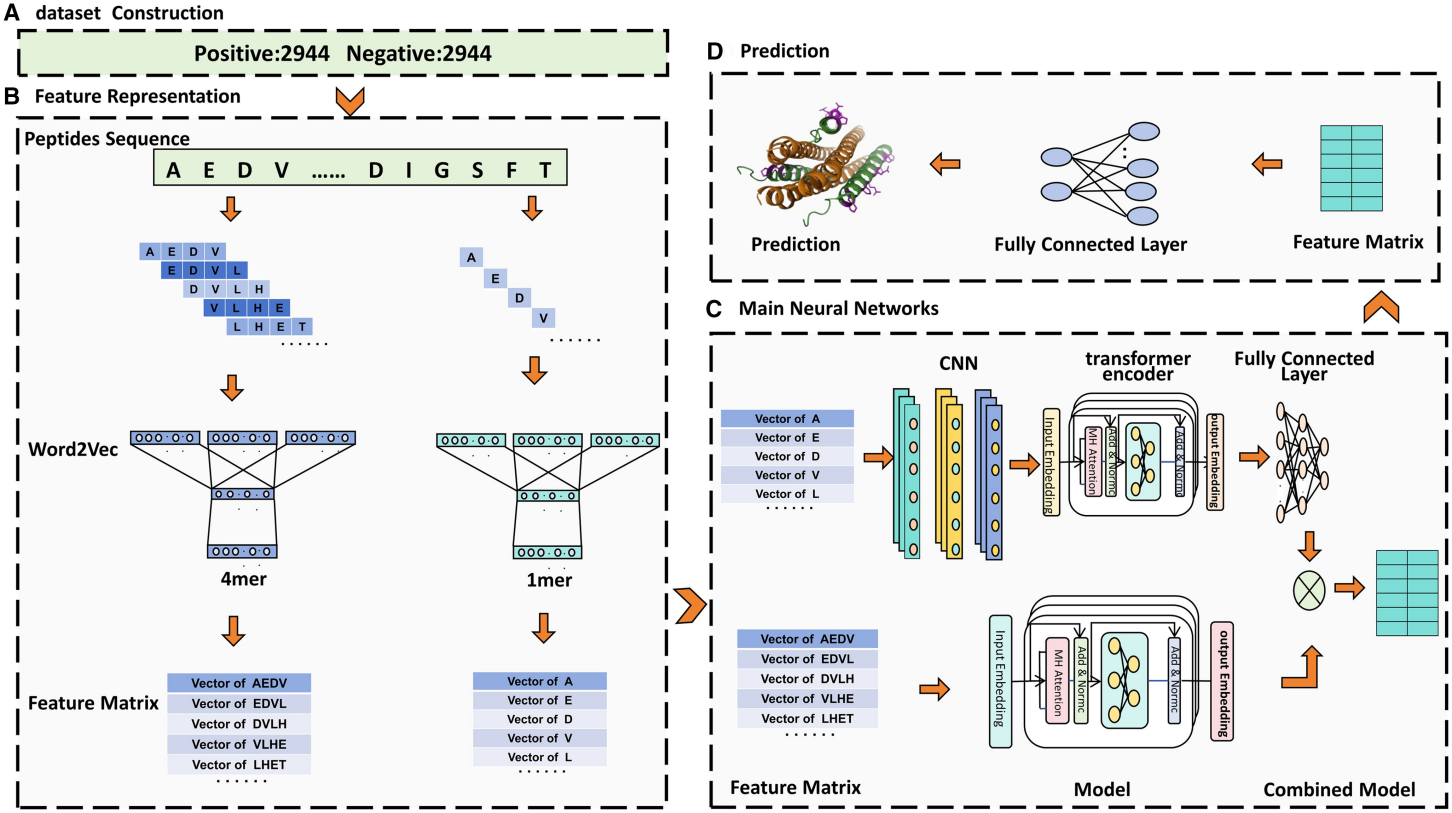
The overall framework of AVPpred-BWR. (A) Dataset construction; (B) feature representation; (C) main neural networks; and (D) prediction.

The C is the window size, $x_{i}\;$is the input vector. The weight matrix V lies between the input and hidden layer. For example, consider a sequence ACEKLHET, our model performs 1mer, 4mer segmentation on it, utilizing a step size of 1, as shown in Fig. 1 and Supplementary Fig. S2.

### 2.3 Proposed model

The workflow of our model is shown in Fig. 1 which mainly includes the following steps: (i) Constructing the datasets and processing the sequences into fixed length. (ii) Feature representation: peptide sequences were represented as natural language sentences with 4mer and 1mer as a word, respectively. These sentences are then fed into the two Word2Vec models to obtain two embedding matrices, respectively. (iii) Main neural networks: the embeddings obtained from the two Word2Vec models are fed into two network channels. The first channel is a hybrid neural networks consisted of three layers of 1D CNN and three layers of Transformer encoder (labeled as Transformer1 for the next sections). And the output of the Transformer encoder is further processed with three dense layers. The second channel consists solely of three layers of Transformer encoders (labeled as Transformer2 for the next sections). (iv) Prediction: the features extracted from the two channels are concatenated and then are inputted to the fully connected network to derive the final prediction probability. The details about the architecture are shown in Table 1. The detailed descriptions of the 1D convolutional neural network and the Transformer encoder can be found in the supplemental materials.

**Table 1.** The architecture, training parameters, and hardware details of our model.

Architecture			Training parameters	Model environment	Hardware
Channel 1	Layers	Input shape	Batch size (32),	Keras = 2.11.0,	NVIDIA
	Conv1D	(None, 100, 150)	epoch (16),	TensoFlow = 2.11.0,	RTX
	Conv1D	(None, 98, 128)	Adam,	Python = 3.8.9	A6000
	Conv1D	(None, 96, 128)	learning rate (0.0001)		GPU
	MaxPooling1D	(None, 94, 256)			
	Dropout	(None, 92, 256)			
	Transformer encoder*3	(None, 92, 256)			
	Flatten	(None, 92, 256)			
	Dense	(None, 23 552)			
	Dropout	(None, 23 552)			
	Dense	(None, 128)			
	Dense	(None, 64)			
Channel 2	Transformer encoder*3	(None, 97, 150)			
	Flatten	(None, 97, 150)			
	Concatenation	(None, 32), (None, 14 550)			
	Dense	(None, 14 582)			
	Dropout	(None, 64)			
	Dense	(None, 64)			

As presented in Table 1, our model is implemented under python3.8.9. The model was training based on Keras 2.11.0 and Tensorflow 2.11.0. One NVIDIA RTX A6000 GPU is leveraged to accelerate the speed of training and testing. The Adam with learning rate of 0.0001 is chosen as the optimizer. The training epoch is set to 16 and the batch size is configured to be 32. We use accuracy (ACC), Sensitivity (SN), Specificity (SP), Matthew’s correlation coefficient (MCC), and the area under the receiver operating characteristic curve (AUC) as the evaluation metrics. Fivefold cross validation was used for model selection and hyperparameters optimization. The details of these evaluation metrics can be found in Supplemental Materials.

## 3 Results

### 3.1 Performance of embedding combinations of different word sizes

To demonstrate the effect of the embeddings of 1mer and 4mer, the different embedding combinations of 1mer and 2mer, 1mer and 3mer, 2mer and 1mer, 2mer, and 3mer and so on were used to build the models. We fed the embeddings of k-mers (*k* = 1, 2, 3, 4) into different channels. For example, we fed the 1mer embeddings into channel1 and separately input the 2mer, 3mer, and 4mer embeddings into channel2. In a similar fashion, we fed the embeddings of 2mer into channel1 and separately fed the embeddings of 3mer and 4mer into channel2. Then, on the other way round, we fed the 1mer embeddings into channel2, and fed 2mer, 3mer, and 4mer into channel1, respectively, and so on.

As shown in Table 2, for the first six combinations, the model based on the combination of 1mer and 4mer is superior to other models according to ACC, SN, MCC, and AUC values. For the second six combinations, the model based on the combination of 4mer and 1mer is also superior to the other models. This proves that the choice of 4mer and 1mer as words for combining can capture more information and yield optimal results. Accordingly, we selected the model based on the combination of 1mer and 4mer as our final model. Note that the embeddings of SkipGram model were also used to build the model, the result (Supplementary Table S2) indicates that the performance of the model based on CBOW model is slightly better than that of SkipGram model. In addition, the one-hot encodings of peptide sequences were utilized as the input for the CNN+Transformer model. Supplementary Table S2 shows the corresponding cross-validation results which indicate that the model is inferior to our model.

**Table 2.** The 5-fold cross validation performance on the training dataset of the models based on combinations of 1mer, 2mer, 3mer, and 4mer representations.

Input of Channel1	Input of Channel2	ACC	SN	SP	MCC	AUC
1mer	2mer	0.797±0.011	0.851±0.035	0.744±0.041	0.605±0.019	0.890±0.006
1mer	3mer	0.870±0.004	0.918±0.014	0.822±0.011	0.744±0.009	0.936±0.003
1mer	4mer	**0.893**±**0.006**	**0.979**±**0.005**	0.807±0.011	**0.798**±**0.011**	**0.961**±**0.003**
2mer	3mer	0.852±0.005	0.937±0.012	0.768±0.010	0.716±0.010	0.931±0.003
2mer	4mer	0.891±0.005	0.933±0.017	0.850±0.020	0.788±0.008	0.954±0.003
3mer	4mer	0.888±0.004	0.948±0.009	**0.828**±**0.011**	0.783±0.007	0.953±0.004
2mer	1mer	0.798±0.012	0.872±0.023	0.732±0.045	0.615±0.020	0.885±0.006
3mer	1mer	0.862±0.005	0.905±0.025	0.819±0.018	0.729±0.012	0.937±0.002
4mer	1mer	0.873±0.008	0.949±0.014	0.797±0.027	0.757±0.012	0.942±0.002
3mer	2mer	0.843±0.012	0.893±0.025	0.794±0.020	0.693±0.025	0.917±0.009
4mer	2mer	0.886±0.005	0.947±0.009	0.824±0.011	0.778±0.009	0.950±0.005
4mer	3mer	0.884±0.002	0.95±0.015	0.819±0.019	0.777±0.003	0.948±0.003

To facilitate understanding, the highest value in each column is shown in bold.

Moreover, we also evaluated the performance of four kinds of kmer words with different strides. The embeddings based on different settings were used as input of CNN+Transformer model. The cross-validation results are shown in Supplementary Table S3, which indicate that words with step size of 1 achieved the best performance for different word sizes, respectively.

### 3.2 Performance of the different neural network framework

Various deep learning networks have been used in AVPs prediction (Li *et al.* 2020, Lin *et al.* 2022, Du *et al.* 2023), in order to choose the optimal model, we analyze the performance of CNN combined with different neural networks such as BiLSTM (Xiao *et al.* 2021), TCN (Raza *et al.* 2023), LSTM (Liao *et al.* 2023), GRU (Shi and Zhang 2022), and BiGRU (Lin *et al.* 2020). Specifically, we used these networks to replace the Transformer modules (Transformer1 and Transformer2) in our model. As shown in Table 3, according to AUC, ACC, and MCC values, the model based on CNN and Transformer is the best. In addition, the models based on other networks also demonstrate commendable performance which demonstrate again the effectiveness of the embeddings combination of 1mer and 4mer.

**Table 3.** The cross validation performance of the models based on different deep neural networks.

Networks	ACC	SN	SP	MCC	AUC
CNN+BiLSTM1+BiLSTM2	0.871± 0.010	0.890±0.041	0.851±0.031	0.750±0.019	0.948±0.004
CNN+TCN1+TCN2	0.815±0.007	0.985±0.004	0.645±0.018	0.671±0.010	0.901±0.007
CNN+LSTM1+LSTM2	0.865±0.016	0.898±0.052	0.832±0.026	0.741±0.030	0.948±0.006
CNN+GRU1+GRU2	0.883±0.005	0.912±0.017	**0.854**±**0.023**	0.770±0.009	0.951±0.006
CNN+BiGRU1+BiGRU2	0.865±0.005	0.903±0.032	0.828±0.035	0.740±0.008	0.943±0.004
CNN+Transformer1+ Transformer2	**0.893**±**0.006**	**0.979**±**0.005**	0.807±0.011	**0.798**±**0.011**	**0.961**±**0.003**

To facilitate understanding, the highest value in each column is shown in bold.

**Figure 2.**
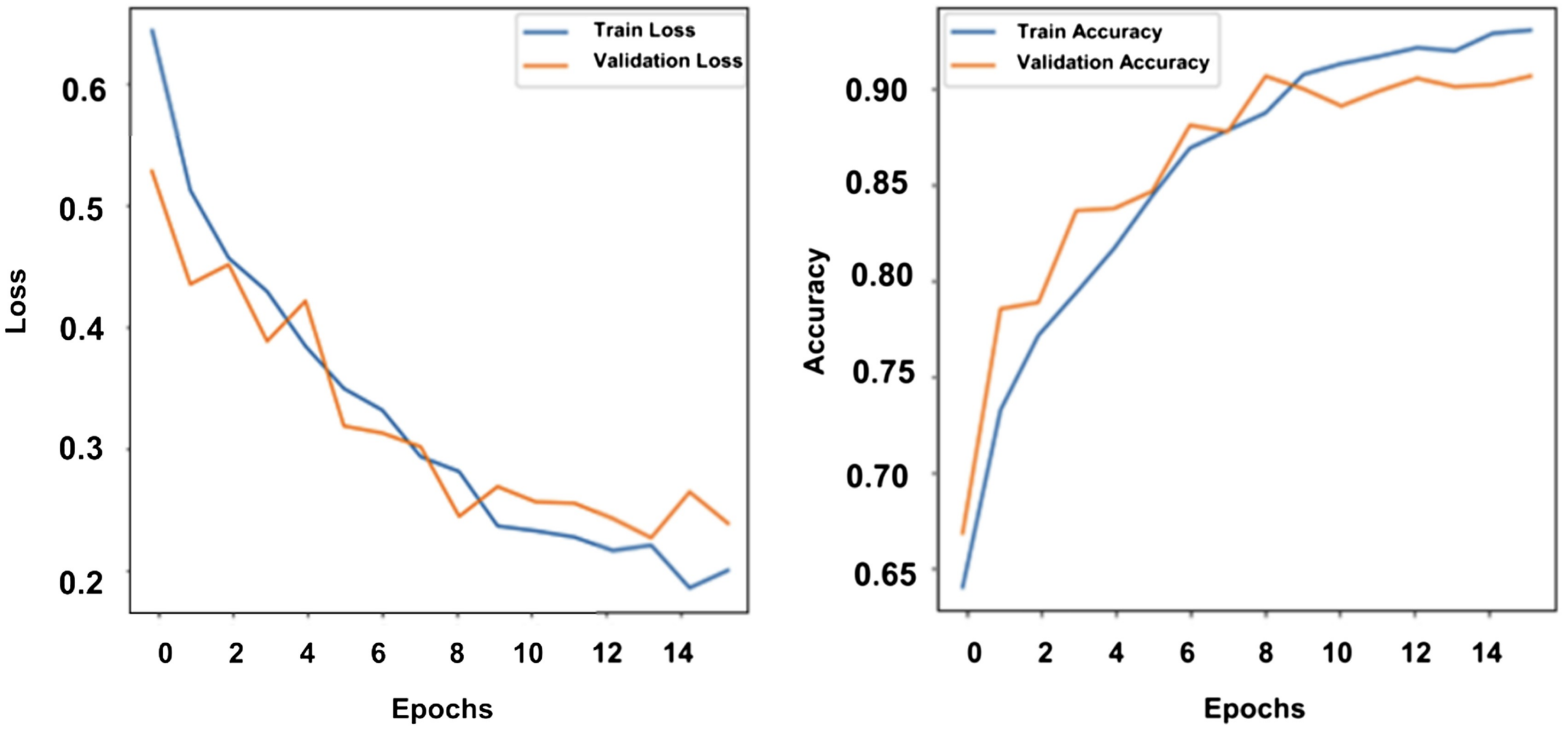
The training convergence curves of our model. The left panel is for validation loss curve. The right panel is for validation accuracy curve.

The architectures, training hyperparameters, and model parameters of these networks were summarized in Supplementary Table S4. Clearly, our model has the largest trainable parameters, to make sure our model was not over-fitted, we plotted the training convergence curve of our model as shown in Fig. 2 which indicates that our model is not over-fitted.

Besides, we also compared our model with the models based on traditional shallow learning algorithms such as support vector machine (SVM), random forest and eXtreme Gradient Boosting (XGBoost). The 5-fold cross-validation results of these models were shown in Supplementary Table S5 which indicate that the models based on shallow learning algorithms also yield promising results which demonstrate the effectiveness of the proposed feature representations. However, the performance of those models is inferior to our deep learning based on model.

### 3.3 Ablation analysis of our model

To analyze the contribution of different modules for our model, we removed the three modules, respectively, to obtain three model variants. In addition, we also generated the other two variants by keeping only CNN of channel1 and by keeping only channel2, respectively. The cross-validation performance on the training dataset of these model variants is shown in Table 4. The architecture of the models could affect the distribution of the predicted probabilities. For example, CNN excels in local pattern recognition thus the corresponding models might achieve high precision and accuracy and the Transformer excels in capture global information such that the corresponding models might achieve high recall and MCC. However, these metrics can change with different thresholds, the AUROC is not changed with different thresholds. Thus, we considered the AUROC values as the most important metric to evaluate different models. Table 4 indicate that the AUC values of the five model variants are all smaller than our original model. Our results illustrate the importance of all the three modules.

**Table 4.** The ablation study results of the models based on different deep networks on training set.

CNN	Transformer1	Transformer2	ACC	SN	SP	MCC	AUC
√	×	×	0.774	0.791	0.757	0.561	0.870
×	√	√	0.896	0.989	0.803	0.807	0.957
√	×	√	0.905	0.950	0.859	0.813	0.955
√	√	×	0.823	0.935	0.710	0.664	0.871
×	×	√	0.888	0.960	0.816	0.787	0.947
√	√	√	0.893	0.979	0.807	0.798	0.961

### 3.4 Comparison with existing methods

**Figure 3.**
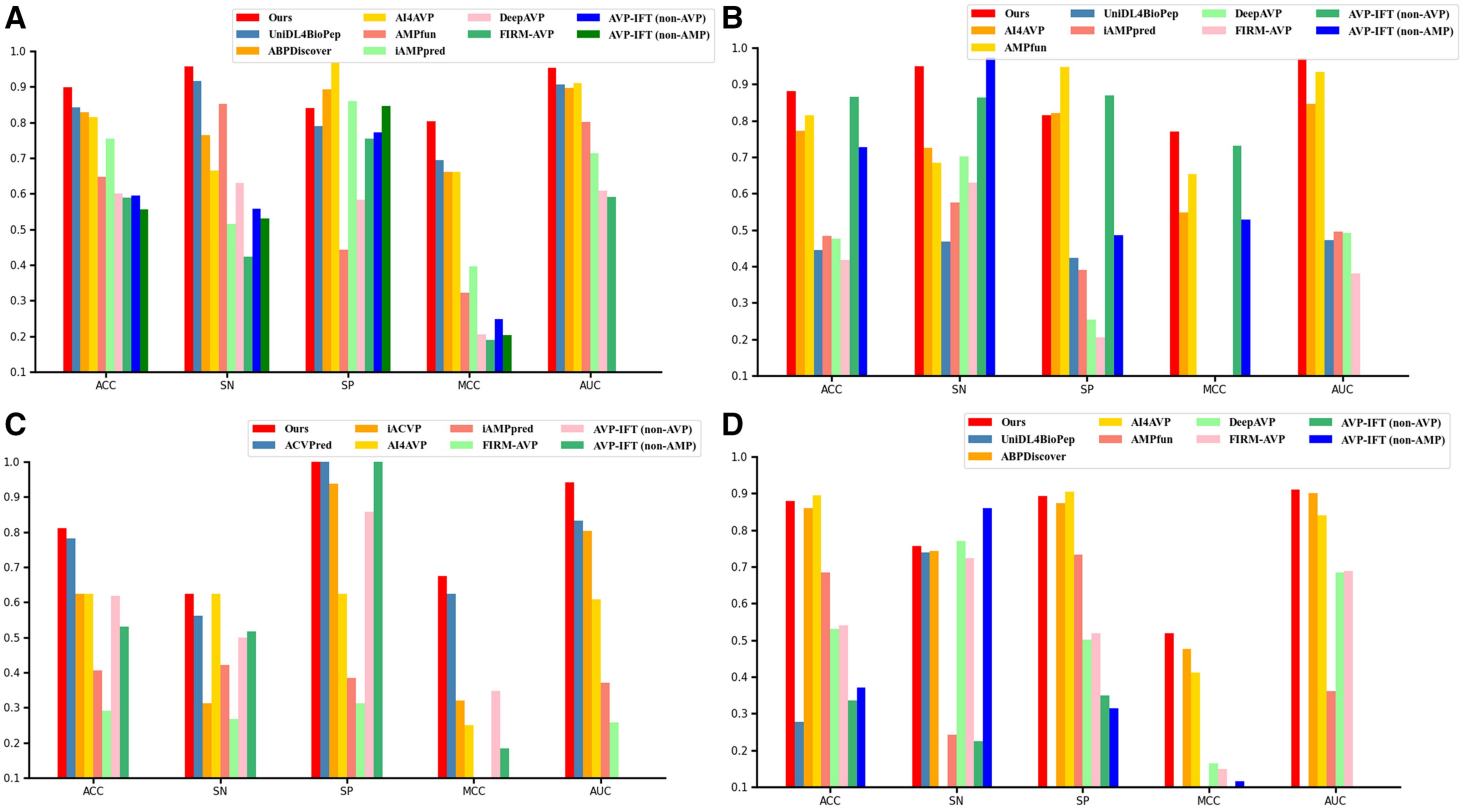
Performance comparison between different models on the independent test set and the other three external test set. (A) the independent test set; (B) the external test set1; (C) the external test set2; and (D) the external test set3.

To validate the generalization of our model, we compared our model with several state-of-the-art models such as UniDL4BioPep (Du *et al.* 2023), ABPDiscover (Pinacho-Castellanos *et al.* 2021), AI4AVP (Lin *et al.* 2022), AVP-IFT (Guan *et al.* 2024), iAMPpred (Meher *et al.* 2017), FIRM-AVP (Chowdhury *et al.* 2020), AMPfun (Chung *et al.* 2020), and DeepAVP (Li *et al.* 2020), on the independent test set. We first summarized the information of those state-of-the-art models in Supplementary Table S6 which shows the differences between our model and other models in three different aspects which are feature representation, feature selection, and model architecture. Figure 3A and Supplementary Table S7 presents the prediction results of different models on the independent test set. Our model achieves an accuracy of 89.9%, MCC of 0.804, and an AUC of 95.3%, with an improvement of 5.7%, 11.0%, and 4.3% compared to the second-high values, respectively, which indicate that our model can perform well in identifying AVPs. The ROC curves of different models were shown in Supplementary Fig. S3.

To further demonstrate the generalization of our model, three external independent test sets were adopted to evaluate the performance. The first external test set contains AVPs collected from updated databases such as DRAMP 4.0 (Ma *et al.* 2025), dbAMP 3.0 (Yao *et al.* 2025), APD3 (Wang *et al.* 2016), DBAASP v3 (Pirtskhalava *et al.* 2021), and so on, which contains 475 positive and negative samples. Figure 3B and Supplementary Table S8 indicate that our model achieves an accuracy of 88.2%, MCC of 0.771 and an AUC of 97.0%, with an improvement of 1.6%, 3.9%, and 3.6% compared to the second-high values, respectively. The second external test set was obtained from Xu *et al.*’s (2024) paper. At the onset of the COVID-19 pandemic, scientists swiftly initiated the search for anti-coronavirus peptides (ACVPs), leading to the discovery of a variety of potential anti-coronavirus peptides (Xu *et al.* 2024). The test set contains 16 positive samples and 16 negative samples. Figure 3C and Supplementary Table S9 demonstrate that our model achieves an AUC of 0.941 which is about 11% higher than the state-of-the-art model ACVPred. The third external test set is an imbalanced dataset containing 1230 positive samples and 10 771 negative samples. Figure 3D and Supplementary Table S10 illustrate that our model achieves an MCC of 0.519 and an AUC of 91.0%, with an improvement of 4.3% and 1.0% compared to the second-high values, respectively. The results on the four independent test sets underscore the effectiveness of our model, highlighting its robustness and the strong generalization in AVP prediction.

Moreover, depending on the specific activities of AVPs, we extended our framework to build models for predicting AVPs against six key viral families (Coronaviridae, Retroviridae, Herpesviridae, Paramyxoviridae, Orthomyxoviridae, Flaviviridae) and eight viruses (FIV, HCV, HIV, HPIV3, HSV1, INFVA, RSV, SARS-CoV) based on the AVPs in our dataset. The results are shown in Supplementary Tables S11 and S12, literally, for 12 of the 14 specific tasks, our models show superior performances compared with the corresponding AVP-IFT models.

### 3.5 Model interpretation

The ability to correctly interpret a prediction model’s output is crucial as it provides insight into how a model may be improved, and supports understanding of the process being modeled (Lundberg and Lee 2017). To validate if our model can uncover underlying sequential patterns of AVPs, we visualized the distribution differences between AVPs and non-AVPs by using Two Sample Logo (Vacic *et al.* 2006), meanwhile, we also visualized the attention weights learned by Transformer encoders of our model.

**Figure 4.**
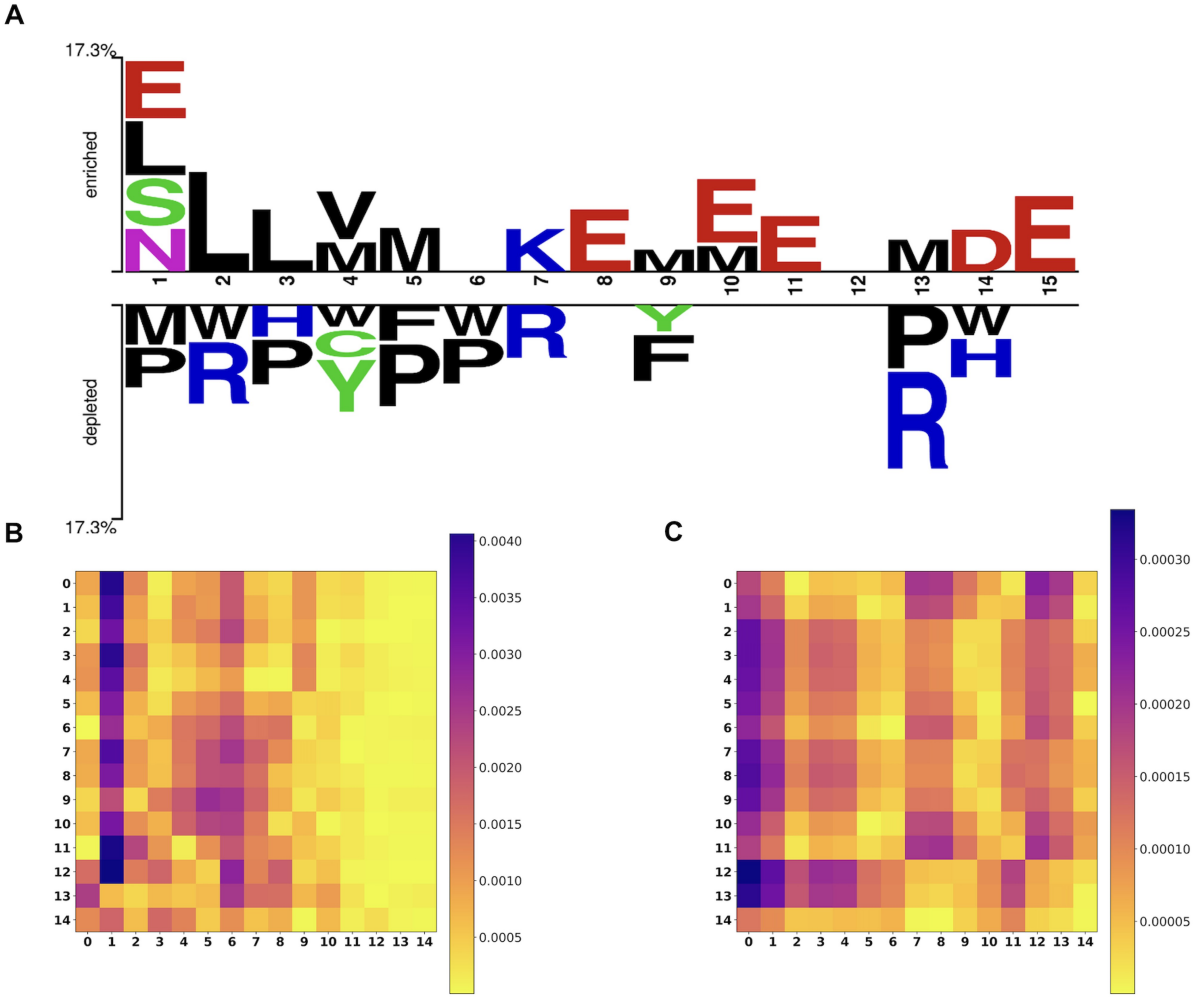
Overall sequence pattern discrepancy between positive and negative samples illustrated by Two Sample Logo (A), and the heatmaps of the attention weights generated by Transformer1 for 1mer (B) and by Transformer2 for 4mer (C).

Specifically, we chose the AVPs and non-AVPs sequences of length 15 as an example to interpret our model. Figure 4A shows the Two Sample Logo of AVPs and non-AVPs, which clearly illustrates that there are significant differences between positive and negative samples at positions 1, 2, and 13. On the other hand, Fig. 4B shows the heatmap of the attention weights generated by Transformer1 for 1mer and Fig. 4C shows the heatmap of the attention weights generated by Transformer2 for 4mer. Figure 4B shows that the position 2 (1 of the *x*-axis) has high attention weights. Figure 4C indicates high attention weights at position 1 (0 of the *x*-axis), and medium high attention weights at positions 2 (1 of the *x*-axis) and 13 (12 of the *x*-axis). Thus, our analysis not only demonstrates the ability of our model to capture the sequential pattern but also demonstrates the complementarity between the two biological words.

Moreover, Fig. 4A also shows that there are three positions (positions 6, 8, 12) at which the differences between positive and negative samples are not significant. However, Fig. 4B indicates that there are medium high attention weights at position 6 and 8 (5 and 7 of the *x*-axis) and Fig. 4C indicates that there are medium high attention weights at the position 12 (11 of the x-axis). These medium-high attention weights at those positions could make the model made error prediction for some examples.

In addition, we checked the metadata of the 26 false positive examples, and found that 22 of them are anti-HIV peptides. Further analysis indicates that most of the 22 peptides are synthetic peptides and are composed of <20 residues.

**Figure 5.**
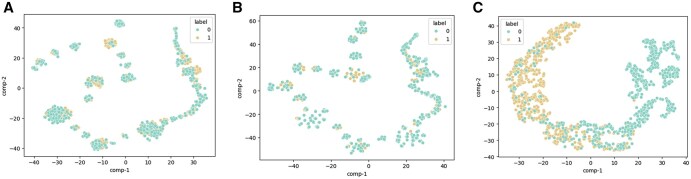
T-SNE plots for feature representations of our model. 0 and 1 represent negative samples and positive samples, respectively. (A) Word2Vec embeddings of 1mer. (B) Word2Vec embeddings of 4mer. (C) The features extracted from the fully connected layer of the prediction module.

Meanwhile, to reveal the representability enhancement during the feature processing of our model, we visualized the input features and features obtained from the fully connected layer by using T-SNE (Arora *et al.* 2018). As shown in Fig. 5, the positive and negative samples from the independent test sets are mixed in the input layer and eventually separated clearly after the fully connected layer of the prediction module.

### 3.6 Transferability of our model

To validate the transferability of the proposed biological words, the proposed pipeline was extended to predict anticancer peptides and DPP-IV inhibitory peptides. The anticancer peptides dataset, comprising 970 positive and negative samples, was collected from AntiCP 2.0 (Agrawal *et al.* 2021). The DPP-IV inhibitory peptide dataset was collected from iDPPIV-SCM, including 665 positive and negative samples (Charoenkwan *et al.* 2020). The length distributions of positive and negative samples of these two kinds of peptides were also shown in Supplementary Fig. S1. The datasets of these two kinds of bioactive peptides were divided into training and independent test sets. The details of DPP-IV inhibitory peptide and ACP datasets are shown in Supplementary Table S1.

Based on the training sets of the two kinds of bioactive peptides, we built the corresponding models by using the same pipeline as we did for AVPs. Supplementary Table S13 shows the cross-validation results on the training datasets. To illustrate the generalization of the two models, the performance of our models was compared with the existing models on the independent test sets. Table 5 shows the predictive results on the two independent test sets, which indicate that our models outperform other existing state-of-the-art models according to the AUC values. These results demonstrate that the effectiveness of the proposed pipeline is largely attributed to the representations of biological words and prove that the choice of four residues and one residue as words for splicing can capture more information and yield optimal results.

**Table 5.** The performance comparison on the two independent test datasets for ACP and DDPIV inhibitory peptide.

Bioactivity	Models^a^	ACC	SN	SP	MCC	AUC
Anticancer peptide	AntiCP 2.0	0.754	**0.774**	0.734	0.510	N/A
	UniDL4BioPep	0.735	0.734	0.737	0.471	0.805
	iACP-FSCM	**0.825**	0.726	**0.903**	**0.646**	0.812
	Ours	0.765	0.733	0.797	0.530	**0.834**
DPP-IV inhibitory peptide	UniDL4BioPep	0.853	0.861	0.846	0.707	0.938
	iDPPIV-SCM	0.797	0.789	0.805	0.594	0.847
	Ours	**0.883**	**0.887**	**0.878**	**0.767**	**0.960**

a The predictive results of AntiCP 2.0, iACP-FSCM, iDPPIV-SCM, and UniDL4BioPep were collected from Du *et al.* (2023). To facilitate understanding, the highest value in each column is shown in bold.

### 3.7 Secondary structures analysis

**Figure 6.**
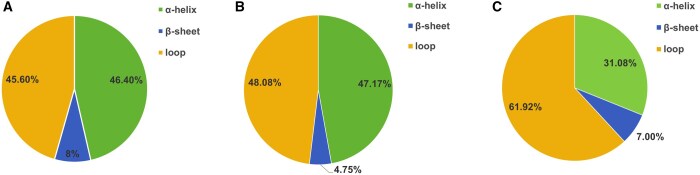
The secondary structure composition of the peptide residues in our datasets. (A) AVP dataset; (B) ACP dataset; and (C) DPP-IV inhibitory peptide dataset.

To further validate the rationale about using 4mer and 1mer to correspond the two kinds of secondary structure, we used JPred4 (Drozdetskiy *et al.* 2015) to predict the secondary structures of the peptides in our datasets and analyze the secondary structure composition. Figure 6 shows the secondary structure compositions of the three datasets. Specifically, for AVP dataset, most of the 118 372 residues belong to α-helixes and loops, reaching 46.4% and 45.6%, respectively. For anticancer peptide dataset, most of 40 644 residues belong to α-helixes and loops, reaching 47.17% and 48.08%, respectively. And for DPP-IV inhibitory peptide dataset, most of 14 468 residues belong to α-helixes and loops, reaching 31.08% and 61.92%, respectively.

## 4 Conclusion

The emergence of AVPs provides new potential means of preventing and treating viral infections, offers promising avenues for exploring novel antiviral therapeutic approaches. Inspired by the secondary structures of peptides, multi-scale representation and multi-granularity semantic analysis, we proposed using biological words to represent peptide sequences as natural language sentences. Word2Vec was used to obtain the embeddings of these biological words, based on which we built our model, AVPpred-BWR. By comparing with other state-of-the-art models, we demonstrate the effectiveness of the proposed biological words. We believe that our model could be an efficient tool for identifying AVPs and the proposed biological words could be an effective representation for bioactive peptides recognition.

Although we have used comprehensive benchmark datasets to build and evaluate our model, the size of the datasets is still not big enough. With more experimental data generating, the model can be updated in the future. Furthermore, we only used the embeddings of the proposed biological words to build our model, considering the complementarity between different kinds sequential features and structural features, multiple view features could be incorporated for model development. Moreover, current work is mainly for differentiating AVPs and non-AVPs, future work involves to build multi-label classification models based on multi-labeled data of AVPs.

## Supplementary Material

btaf126_Supplementary_Data

## Data Availability

The data underlying this article are available at: https://github.com/zyweizm/AVPpred-BWR or https://zenodo.org/records/14880447 (doi: 10.5281/zenodo.14880447).
