# Supplementary material for: AVPpred-BWR: antiviral peptides prediction via biological words representation
Source: Bioinformatics. 2025 Mar 27;41(4):btaf126. doi: 10.1093/bioinformatics/btaf126 (PMC11968319; doi:10.1093/bioinformatics/btaf126)
Supplement: btaf126_Supplementary_Data [file btaf126_supplementary_data.zip › Supplementary materials for AVPpred-BWR_02162025.docx]

**Supplementary Materials for:**

**AVPpred-BWR: Antiviral Peptides Prediction via Biological Words Representation**

Zhuoyu Wei 1,#, Yongqi Shen 1,#, Xiang Tang 1, Jian Wen 1, Youyi Song 2, Mingqiang Wei 3, Jing Cheng 1,* and Xiaolei Zhu 1,*

1 School of Information and Artificial Intelligence, Anhui Agricultural University, Hefei, Anhui, 230036, China

2 School of Science, China Pharmaceutical University, Nanjing, 210009, China

3 School of Computer Science and Technology, Nanjing University of Aeronautics and Astronautics, Nanjing, 210016, China

* Corresponding authors: [xlzhu_mdl@hotmail.com](mailto:xlzhu_mdl@hotmail.com); [chengjing@ahau.edu.cn](mailto:chengjing@ahau.edu.cn)；

# Joint first authors

**Convolutional Neural Network**

Convolutional Neural Network (CNN) is a powerful class of deep learning frameworks widely applied in various tasks, including object detection, speech recognition, computer vision, image classification, and bioinformatics (Tasdelen and Sen, 2021). The major components of CNN include the convolutional layer, pooling layer, and fully connected layer (Lu, et al., 2021). It can automatically learn and recognize features from the data without manually extracting features. In this study, the input matrix sizes for the CNN layer are (100,150), which represents the features of 1mer biological words via Word2Vec. Three layers of convolutional neural network are used in our model and normalization layer and dropout layer are added to accelerate the training process to stabilize the model and prevent overfitting. They are activated by using the ReLU activation function because the ReLU activation function is sparse which reduces the possibility of gradient disappearance compared to the sigmoid function. The ReLU activation function is defined by:

, (4)

where, is the output of the ReLU activation function, and the dropout ratio is set to 0.2. After being processed by the CNN neural network, the features are then fed into the Transformer encoder layer.

**Transformer encoder**

The Transformer encoder encodes the input sequence through components such as multi-head attention mechanism, residual join, layer normalization and feed-forward neural network (Guntuboina, et al., 2023). Transformer encoder is able to capture the information in the sequence at different locations and points of attention, while ensuring training stability through residual concatenation and normalization. The use of multi-head attention mechanism is similar to the use of multiple convolutional kernels in the same convolutional layer in CNN. This enhancement allows the model to tokenize different features in different subspaces and avoids the suppression of those features by average pooling.

Multi-head attention mechanism is composed of multiple distinct self-attention in parallel, as shown in Figure S4. Given an input sequence , the output of the self-attention function is a sequence of weighted sums , where each is a weighted sum of the input sequence. This weighted sum is obtained by performing a weighted summation over all input locations, with weights determined by the attention scores. The self-attention function is given below:

, (5)

where, is the Query Matrix, is the Key Matrix, is the Value Matrix, is the dimensions of the query and the key for scaling the Attention Score. The attention scores are obtained by computing the dot product between the query and the key and dividing by for scaling. Finally, these weights are applied to the value matrix to obtain the weighted sum, which serves as the output of the self-attention function. These components help to encode the information of the input sequence and motivate the model to well capture the relationships in the sequence.

The 1mer embeddings processed by CNN and Transformer hybrid neural network feature extraction is combined with the 4mer embeddings processed by CNN feature extraction. The extracted representations from the two channels were concatenated and fed into a fully-connected layer to do the final prediction through a Softmax activation function. The Softmax activation function is defined by:

, (6)

is the output of the Softmax function, and the result interval is (0,1).

**Model evaluation**

We used MCC (Matthew’s correlation coefficient), SP (Specificity), SN (Sensitivity), ACC (Accuracy), and AUC (Area Under the receiver operating characteristic Curve) as the evaluation metrics. They are calculated by the following formulas:

, (7)

, (8)

, (9)
 , (10)

Where (True Positive) is positive sample predicted by the model to be in the positive class. (True Negative) is negative sample predicted by the model to be in the negative class. (False Positive) is negative samples predicted by the model to be positive. (False Negative) is positive sample predicted by the model to be in the negative class. The is the area under the Receiver Operating Characteristic Curve.

**Table S1****.** The summary of the datasets used in this study

| Bioactive peptides | Datasets | # of Positive | # of Negative |
| --- | --- | --- | --- |
| Antiviral Peptide | Train set | 2321 | 2321 |
|  | Test set | 623 | 623 |
|  | External test set1 | 475 | 475 |
|  | External test set2 | 16 | 16 |
|  | External test set3 | 1230 | 10771 |
| Anticancer Peptide | Train | 689 | 689 |
|  | Test | 172 | 172 |
| DPP-IV inhibitory peptide | Train | 532 | 532 |
|  | Test | 133 | 133 |

**Table S2**. The 5-fold cross validation performance on the training dataset of the different embedding methods.

| Embedding methods | ACC | SN | SP | MCC | AUC |
| --- | --- | --- | --- | --- | --- |
| One-hot coding | 0.834 | 0.841 | 0.827 | 0.671 | 0.912 |
| Skip-gram | 0.893 | 0.963 | 0.823 | 0.795 | 0.959 |
| CBOW(ours) | 0.893 | 0.979 | 0.807 | 0.798 | 0.961 |

**Table S3.** The cross-validation results of the models based on different strides of different kmers.

| Models | ACC | SN | SP | MCC | AUC |
| --- | --- | --- | --- | --- | --- |
| 1mer (stride = 1) | 0.8 | 0.866 | 0.735 | 0.608 | 0.885 |
| 2mer(stride = 1) | 0.787 | 0.697 | 0.878 | 0.591 | 0.898 |
| 2mer(stride = 2) | 0.781 | 0.808 | 0.755 | 0.573 | 0.867 |
| 3mer(stride = 1) | 0.825 | 0.803 | 0.847 | 0.653 | 0.912 |
| 3mer(stride = 2) | 0.775 | 0.824 | 0.726 | 0.556 | 0.855 |
| 3mer(stride = 3) | 0.706 | 0.818 | 0.595 | 0.429 | 0.792 |
| 4mer(stride = 1) | 0.866 | 0.939 | 0.792 | 0.744 | 0.937 |
| 4mer(stride = 2) | 0.803 | 0.901 | 0.704 | 0.625 | 0.894 |
| 4mer(stride = 3) | 0.715 | 0.916 | 0.514 | 0.482 | 0.803 |
| 4mer(stride = 4) | 0.762 | 0.906 | 0.618 | 0.554 | 0.833 |

**Table S4**. The numbers of parameters of different network architectures

| Model | Architecture | | | Trainable | Non-trainable  parameters |
| --- | --- | --- | --- | --- | --- |
|  |  |  |  | parameters |  |
| CNN+ BiLSTM1+ BiLSTM2 | Channel 1 | layers | Input shape | 5,502,626 | 512 |
|  |  | Conv1D | (None,100, 150) |  |  |
|  |  | Conv1D | (None, 98, 128) |  |  |
|  |  | Conv1D | (None, 96, 128) |  |  |
|  |  | MaxPooling1D | (None, 94, 256) |  |  |
|  |  | Dropout | (None, 92, 256) |  |  |
|  |  | BiLSTM*3 | (None, 92, 256) |  |  |
|  |  | Flatten | (None, 92, 256) |  |  |
|  |  | Dense | (None, 23552) |  |  |
|  |  | Dropout | (None, 23552) |  |  |
|  |  | Dense | (None, 128) |  |  |
|  |  | Dense | (None, 64) |  |  |
|  | Channel 2 | BiLSTM*3 | (None, 97, 150) |  |  |
|  |  | Flatten | (None, 97, 256) |  |  |
|  |  | Concatenation | (None,32), (None, 24832) |  |  |
|  |  | Dense | (None, 24864) |  |  |
|  |  | Dropout | (None, 64) |  |  |
|  |  | Dense | (None, 64) |  |  |
| CNN+ TCN1+ TCN2 | Channel 1 | layers | Input shape | 4945282 | 1024 |
|  |  | Conv1D | (None,100, 150) |  |  |
|  |  | Conv1D | (None, 98, 128) |  |  |
|  |  | Conv1D | (None, 96, 128) |  |  |
|  |  | MaxPooling1D | (None, 94, 256) |  |  |
|  |  | Dropout | (None, 92, 256) |  |  |
|  |  | TCN*3 | (None, 92, 256) |  |  |
|  |  | Flatten | (None, 92, 128) |  |  |
|  |  | Dense | (None, 117762) |  |  |
|  |  | Dropout | (None, 11776) |  |  |
|  |  | Dense | (None, 128) |  |  |
|  |  | Dense | (None, 64) |  |  |
|  | Channel 2 | TCN*3 | (None, 97, 150) |  |  |
|  |  | Flatten | (None, 97, 128) |  |  |
|  |  | Concatenation | (None,32), (None, 12416) |  |  |
|  |  | Dense | (None, 12448) |  |  |
|  |  | Dropout | (None, 64) |  |  |
|  |  | Dense | (None, 64) |  |  |
| CNN+ LSTM1+ LSTM2 | Channel 1 | layers | Input shape | 4043070 | 512 |
|  |  | Conv1D | (None,100, 150) |  |  |
|  |  | Conv1D | (None, 98, 128) |  |  |
|  |  | Conv1D | (None, 96, 128) |  |  |
|  |  | MaxPooling1D | (None, 94, 256) |  |  |
|  |  | Dropout | (None, 92, 256) |  |  |
|  |  | LSTM*3 | (None, 92, 256) |  |  |
|  |  | Flatten | (None, 92, 128) |  |  |
|  |  | Dense | (None, 128) |  |  |
|  |  | Dropout | (None, 117762) |  |  |
|  |  | Dense | (None, 128) |  |  |
|  |  | Dense | (None, 64) |  |  |
|  | Channel 2 | LSTM*3 | (None, 97, 150) |  |  |
|  |  | Flatten | (None, 97, 128) |  |  |
|  |  | Concatenation | (None,32), (None, 12416) |  |  |
|  |  | Dense | (None, 12448) |  |  |
|  |  | Dropout | (None, 64) |  |  |
|  |  | Dense | (None, 64) |  |  |
| CNN+ GRU1+ GRU2 | Channel 1 | layers | Input shape | 3699330 | 1024 |
|  |  | Conv1D | (None,100, 150) |  |  |
|  |  | Conv1D | (None, 98, 128) |  |  |
|  |  | Conv1D | (None, 96, 128) |  |  |
|  |  | MaxPooling1D | (None, 94, 256) |  |  |
|  |  | Dropout | (None, 92, 256) |  |  |
|  |  | GRU*3 | (None, 92, 256) |  |  |
|  |  | Flatten | (None, 92, 128) |  |  |
|  |  | Dense | (None, 11776) |  |  |
|  |  | Dropout | (None, 11776) |  |  |
|  |  | Dense | (None, 128) |  |  |
|  |  | Dense | (None, 64) |  |  |
|  | Channel 2 | GRU*3 | (None, 97, 150) |  |  |
|  |  | Flatten | (None, 97, 128) |  |  |
|  |  | Concatenation | (None,32), (None, 12416) |  |  |
|  |  | Dense | (None, 12448) |  |  |
|  |  | Dropout | (None, 64) |  |  |
|  |  | Dense | (None, 64) |  |  |
| CNN+ BiGRU1+ BiGRU2 | Channel 1 | layers | Input shape | 5649726 | 512 |
|  |  | Conv1D | (None,100, 150) |  |  |
|  |  | Conv1D | (None, 98, 128) |  |  |
|  |  | Conv1D | (None, 96, 128) |  |  |
|  |  | MaxPooling1D | (None, 94, 256) |  |  |
|  |  | Dropout | (None, 92, 256) |  |  |
|  |  | BiGRU*3 | (None, 92, 256) |  |  |
|  |  | Flatten | (None, 92, 256) |  |  |
|  |  | Dense | (None, 23552) |  |  |
|  |  | Dropout | (None, 23552) |  |  |
|  |  | Dense | (None, 128) |  |  |
|  |  | Dense | (None, 64) |  |  |
|  | Channel 2 | BiGRU*3 | (None, 97, 150) |  |  |
|  |  | Flatten | (None, 97, 256) |  |  |
|  |  | Concatenation | (None,32), (None, 24832) |  |  |
|  |  | Dense | (None, 24864) |  |  |
|  |  | Dropout | (None, 64) |  |  |
|  |  | Dense | (None, 64) |  |  |
| CNN+ Transformer1+ Transformer2 | Channel 1 | layers | Input shape | 7723326 | 512 |
|  |  | Conv1D | (None,100, 150) |  |  |
|  |  | Conv1D | (None, 98, 128) |  |  |
|  |  | Conv1D | (None, 96, 128) |  |  |
|  |  | MaxPooling1D | (None, 94, 256) |  |  |
|  |  | Dropout | (None, 92, 256) |  |  |
|  |  | Transformer encoder*3 | (None, 92, 256) |  |  |
|  |  | Flatten | (None, 92, 256) |  |  |
|  |  | Dense | (None, 23552) |  |  |
|  |  | Dropout | (None, 23552) |  |  |
|  |  | Dense | (None, 128) |  |  |
|  |  | Dense | (None, 64) |  |  |
|  | Channel 2 | Transformer encoder*3 | (None, 97, 150) |  |  |
|  |  | Flatten | (None, 97, 150) |  |  |
|  |  | Concatenation | (None,32), (None, 14550) |  |  |
|  |  | Dense | (None, 14582) |  |  |
|  |  | Dropout | (None, 64) |  |  |
|  |  | Dense | (None, 64) |  |  |

**Table S5**. The comparation with models based on shallow baseline learning algorithms.

| Models | Best hyperparameters | ACC | SN | SP | MCC | AUC | AUC  (p-values) |
| --- | --- | --- | --- | --- | --- | --- | --- |
| Ours | epoch; 16,  batchsize: 32 | **0.893** | **0.979** | **0.807** | **0.798** | **0.961** | **----** |
| XGBoost-Baseline | learning_rate: 0.05, max_depth: 6, n_estimators: 100 | 0.887 | 0.960 | 0.814 | 0.783 | 0.948 | 0.006 |
| RF-Baseline | max_depth: None, min_samples_split: 2, n_estimators: 150 | 0.881 | 0.958 | 0.802 | 0.771 | 0.944 | 0.004 |
| SVM-Baseline | C: 1, kernel: linear | 0.797 | 0.851 | 0.744 | 0.605 | 0.901 | 7.33e-05 |

**Table S6.** Comparison and overview of AVPpred-BWR and the state-of-the-art models for binary classification in AVP dataset.

| Models | Peptide representation | Feature selection methods | Model development | References |
| --- | --- | --- | --- | --- |
| UniDL4BioPep | Protein language model (ESM-2) | None | CNN model with six layers | (Du, et al., 2023) |
| ABPDiscover | Generated by ProtDCal | Six feature selection methods (correlation subset, Relief-F, information gain, gain ratio and symmetrical uncertainty) | RF | (Pinacho-Castellanos, et al., 2021) |
| AI4AVP | Generated by the protein-encoding method PC6 and the descriptor encoding | None | CNN and GAN | (Lin, et al., 2022) |
| iAMPpred | AAC, NAAC, PAAC, α-helix propensity, β-sheet propensity, turn propensity, PI, Turn propensity, Net-charge | Information Gain | SVM | (Meher, et al., 2017) |
| DeepAVP | Generated by the One-hot encode | None | CNN and LSTM | (Li, et al., 2020) |
| FIRM-AVP | AAC, DC, PseAA, APseAAC, CTD, DSSP | Three feature selection methods (PPMCCt, MDGI, RFE) | SVM | (Chowdhury, et al., 2020) |
| AVP-IFT | Generated by Binary, BLOSUM62 and Zscale | None | Multi-scale CNN, BiLSTM, Transformer | (Guan, et al., 2024) |
| AMPfun | NCB, NTB, MB, NCC, NTC, MC, AAC, CTDD | Three feature selection methods (CART,Gini importance, SFS) | RF | (Chung, et al., 2020) |
| Ours | Generated by Biological Words Representation | None | CNN and Transformer | This work |

**Table S7.** Comparison with other state-of-the-art models on the independent test set.

| Models a | ACC | SN | SP | MCC | AUC b |
| --- | --- | --- | --- | --- | --- |
| UniDL4BioPep | 0.842 | 0.916 | 0.790 | 0.694 | 0.907 |
| ABPDiscover | 0.828 | 0.764 | 0.892 | 0.662 | 0.896 |
| AI4AVP | 0.815 | 0.664 | 0.966 | 0.661 | 0.910 |
| AMPfun | 0.647 | 0.852 | 0.443 | 0.323 | 0.802 |
| iAMPpred | 0.755 | 0.515 | 0.860 | 0.396 | 0.714 |
| DeepAVP | 0.600 | 0.629 | 0.583 | 0.205 | 0.609 |
| FIRM-AVP | 0.589 | 0.424 | 0.754 | 0.189 | 0.591 |
| AVP-IFT (non-AVP) | 0.594 | 0.557 | 0.772 | 0.249 | N/A |
| AVP-IFT (non-AMP) | 0.555 | 0.530 | 0.847 | 0.203 | N/A |
| Ours | **0.899** | **0.958** | 0.841 | **0.804** | **0.953** |

a The predictive results of ABPDiscover were collected from (Pinacho-Castellanos, et al., 2021). The results of AVP-IFT (non-AVP/non-AMP), iAMPpred, AMPfun and DeepAVP were obtained by the corresponding webservers. And the results of UniDL4BioPep, AI4AVP, and FIRM-AVP were obtained by implementing the source codes. b The webserver of AVP-IFT does not output the predicted probabilities, so the AUC cannot be calculated.

**Table S8.** Comparison with other state-of-the-art models on the first external independent test set.

| Models a | ACC | SN | SP | MCC | AUC b |
| --- | --- | --- | --- | --- | --- |
| UniDL4BioPep | 0.445 | 0.468 | 0.423 | -0.097 | 0.473 |
| AI4AVP | 0.773 | 0.726 | 0.821 | 0.549 | 0.847 |
| AMPfun | 0.815 | 0.684 | 0.947 | 0.654 | 0.934 |
| iAMPpred | 0.483 | 0.575 | 0.391 | -0.035 | 0.495 |
| DeepAVP | 0.476 | 0.701 | 0.253 | -0.052 | 0.492 |
| FIRM-AVP | 0.418 | 0.629 | 0.206 | -0.181 | 0.380 |
| AVP-IFT (non-AVP) | 0.866 | 0.863 | 0.869 | 0.732 | N/A |
| AVP-IFT (non-AMP) | 0.728 | 0.973 | 0.486 | 0.529 | N/A |
| Ours | 0.882 | 0.950 | 0.815 | 0.771 | 0.970 |
| Ours（thred0.6） | 0.916 | 0.943 | 0.888 | 0.83 | 0.970 |
| Ours（thred0.7） | 0.925 | 0.928 | 0.922 | 0.851 | 0.970 |
| Ours（thred0.9） | 0.941 | 0.901 | 0.981 | 0.885 | 0.970 |

a The results of AVP-IFT (non-AVP/non-AMP), iAMPpred, AMPfun and DeepAVP were obtained by the corresponding webservers. And the results of UniDL4BioPep, AI4AVP, and FIRM-AVP were obtained by implementing the source codes. b The webserver of AVP-IFT does not output the predicted probabilities, so the AUC cannot be calculated.

**Table S9**. Comparison with other state-of-the-art models on the second external independent test set.

| Models a | ACC | SN | SP | MCC | AUC |
| --- | --- | --- | --- | --- | --- |
| ACVPred | 0.781 | 0.562 | 1.00 | 0.625 | 0.832 |
| iACVP | 0.625 | 0.312 | 0.937 | 0.320 | 0.804 |
| AI4AVP | 0.625 | 0.625 | 0.625 | 0.250 | 0.608 |
| iAMPpred | 0.406 | 0.421 | 0.385 | -0.191 | 0.371 |
| FIRM-AVP | 0.290 | 0.267 | 0.313 | -0.420 | 0.258 |
| AVP-IFT (non-AVP) | 0.619 | 0.500 | 0.857 | 0.347 | N/A |
| AVP-IFT (non-AMP) | 0.531 | 0.516 | 1.00 | 0.183 | N/A |
| Ours | **0.812** | **0.625** | 1.00 | **0.674** | **0.941** |

a The predictive results of ACVPred and iACP were collected from (Xu, et al., 2024). The results of AVP-IFT (non-AVP/non-AMP), and iAMPpred were obtained by the corresponding webservers. And the results of AI4AVP and FIRM-AVP were obtained by implementing the source codes.

**Table S10**. Comparison with other state-of-the-art models on the third external independent test set.

| Models a | ACC | SN | SP | MCC | AUC b |
| --- | --- | --- | --- | --- | --- |
| UniDL4BioPep | 0.277 | 0.739 | 0.0113 | -0.396 | 0.0648 |
| ABPDiscover | 0.860 | 0.742 | 0.873 | 0.476 | 0.900 |
| AI4AVP | 0.895 | 0.095 | 0.904 | 0.411 | 0.841 |
| AMPfun | 0.684 | 0.242 | 0.734 | -0.015 | 0.362 |
| DeepAVP | 0.530 | 0.770 | 0.502 | 0.165 | 0.685 |
| FIRM-AVP | 0.540 | 0.724 | 0.519 | 0.148 | 0.689 |
| AVP-IFT (non-AVP) | 0.336 | 0.224 | 0.349 | -0.266 | N/A |
| AVP-IFT (non-AMP) | 0.371 | 0.860 | 0.315 | 0.116 | N/A |
| Ours | 0.879 | 0.757 | 0.892 | 0.519 | 0.910 |
| Ours (thred0.6) | 0.891 | 0.747 | 0.908 | 0.542 | 0.910 |
| Ours (thred0.8) | 0.909 | 0.694 | 0.934 | 0.566 | 0.910 |

a The predictive results of ABPDiscover were collected from (Pinacho-Castellanos, et al., 2021). The results of AVP-IFT (non-AVP/non-AMP), AMPfun and DeepAVP were obtained by the corresponding webservers. And the results of UniDL4BioPep, AI4AVP, and FIRM-AVP were obtained by implementing the source codes. b The webserver of AVP-IFT does not output the predicted probabilities, so the AUC cannot be calculated.

**Table S11.** The predictive results on the independent test sets of the six viral families.

| datasets | ACC | SN | SP | MCC | AUC |
| --- | --- | --- | --- | --- | --- |
| Coronaviridae (our-model) | 0.986 | 0.631 | 0.998 | 0.757 | 0.967 |
| Coronaviridae (AVP-IFT) | 0.898 | 0.935 | 0.895 | 0.571 | N/A |
|  |  |  |  |  |  |
| Retroviridae (our-model) | 0.871 | 0.819 | 0.893 | 0.700 | 0.927 |
| Retroviridae (AVP-IFT) | 0.901 | 0.920 | 0.890 | 0.795 | N/A |
|  |  |  |  |  |  |
| Herpesviridae (our-model) | 0.936 | 0.792 | 0.948 | 0.642 | 0.922 |
| Herpesviridae (AVP-IFT) | 0.848 | 0.881 | 0.845 | 0.520 | N/A |
|  |  |  |  |  |  |
| Paramyxoviridae (our-model) | 0.983 | 0.815 | 1 | 0.894 | 0.976 |
| Paramyxoviridae (AVP-IFT) | 0.963 | 0.912 | 0.968 | 0.815 | N/A |
|  |  |  |  |  |  |
| Orthomyxoviridae (our-model) | 0.986 | 0.727 | 0.997 | 0.797 | 0.983 |
| Orthomyxoviridae (AVP-IFT) | 0.804 | 0.966 | 0.796 | 0.365 | N/A |
|  |  |  |  |  |  |
| Flaviviridae (our-model) | 0.958 | 0.766 | 0.994 | 0.835 | 0.955 |
| Flaviviridae (AVP-IFT) | 0.838 | 0.975 | 0.807 | 0.641 | N/A |

**Table S12.** The predictive results on the independent test sets of targeted viruses.

| datasets | ACC | SN | SP | MCC | AUC |
| --- | --- | --- | --- | --- | --- |
| FIV (our-model) | 0.958 | 0.500 | 0.974 | 0.426 | 0.906 |
| FIV (AVP-IFT) | 0.856 | 0.920 | 0.853 | 0.386 | N/A |
|  |  |  |  |  |  |
| HIV (our-model) | 0.871 | 0.795 | 0.897 | 0.673 | 0.93 |
| HIV (AVP-IFT) | 0.882 | 0.824 | 0.909 | 0.729 | N/A |
|  |  |  |  |  |  |
| HCV (our-model) | 0.969 | 0.810 | 0.996 | 0.871 | 0.983 |
| HCV (AVP-IFT) | 0.844 | 0.882 | 0.837 | 0.591 | N/A |
|  |  |  |  |  |  |
| HPIV3 (our-model) | 1 | 1 | 1 | 1 | 1 |
| HPIV3 (AVP-IFT) | 0.964 | 1 | 0.963 | 0.679 | N/A |
|  |  |  |  |  |  |
| HSV1 (our-model) | 0.998 | 0.944 | 1 | 0.971 | 0.999 |
| HSV1 (AVP-IFT) | 0.845 | 0.963 | 0.835 | 0.516 | N/A |
|  |  |  |  |  |  |
| INFVA (our-model) | 0.986 | 0.636 | 1 | 0.792 | 0.941 |
| INFVA (AVP-IFT) | 0.850 | 0.964 | 0.845 | 0.413 | N/A |
|  |  |  |  |  |  |
| RSV (our-model) | 0.989 | 0.750 | 1.00 | 0.861 | 0.985 |
| RSV(AVP-IFT) | 0.941 | 0.999 | 0.938 | 0.638 | N/A |
|  |  |  |  |  |  |
| SARS-CoV (our-model) | 0.989 | 0.722 | 0.998 | 0.814 | 0.963 |
| SARS-CoV (AVP-IFT) | 0.898 | 0.886 | 0.899 | 0.501 | N/A |

**Table S13.** The cross-validation performance of AVPpred_RBW framework on the training sets of the other two bioactive peptides.

| Bioactive peptides | ACC | SN | SP | MCC | AUC |
| --- | --- | --- | --- | --- | --- |
| Anticancer Peptide | 0.915 | 0.926 | 0.903 | 0.830 | 0.949 |
| DPP-IV inhibitory peptide | 0.949 | 0.955 | 0.943 | 0.899 | 0.984 |


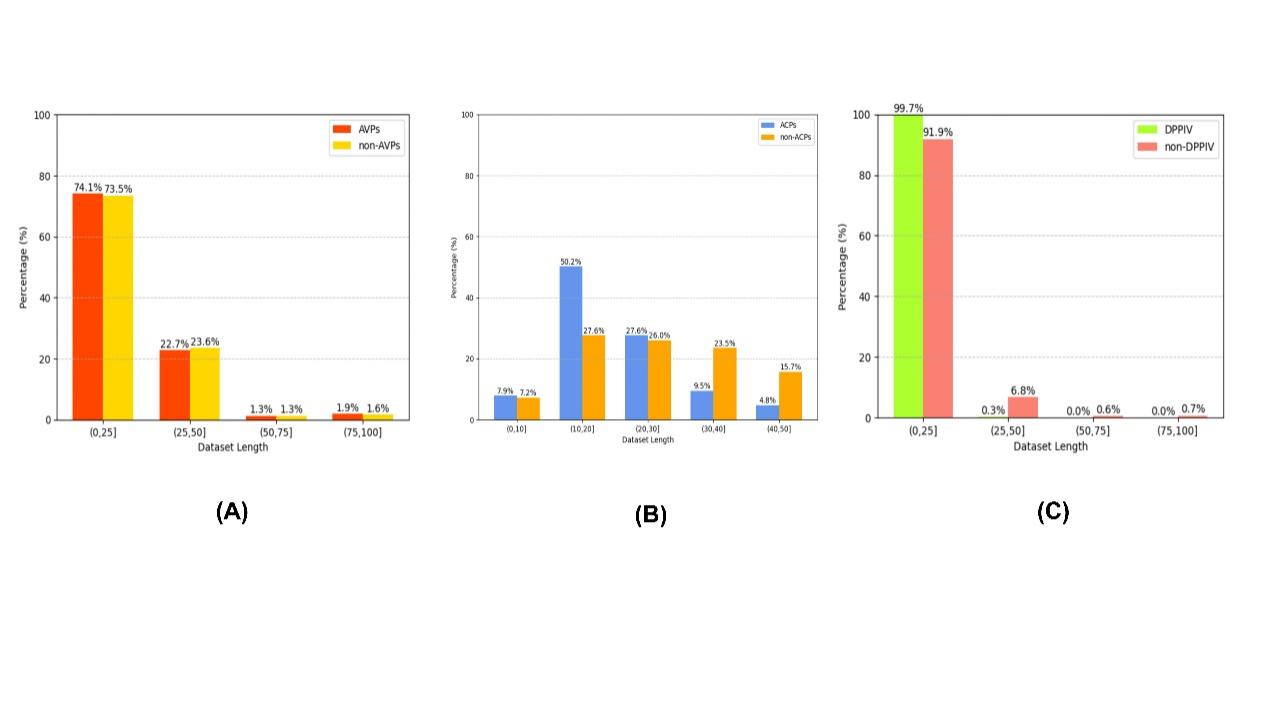


**Figure S1.** The length distribution of positive and negative samples in our datasets. A: AVP dataset; B: ACP dataset; C: DPP-IV inhibitory peptide dataset.


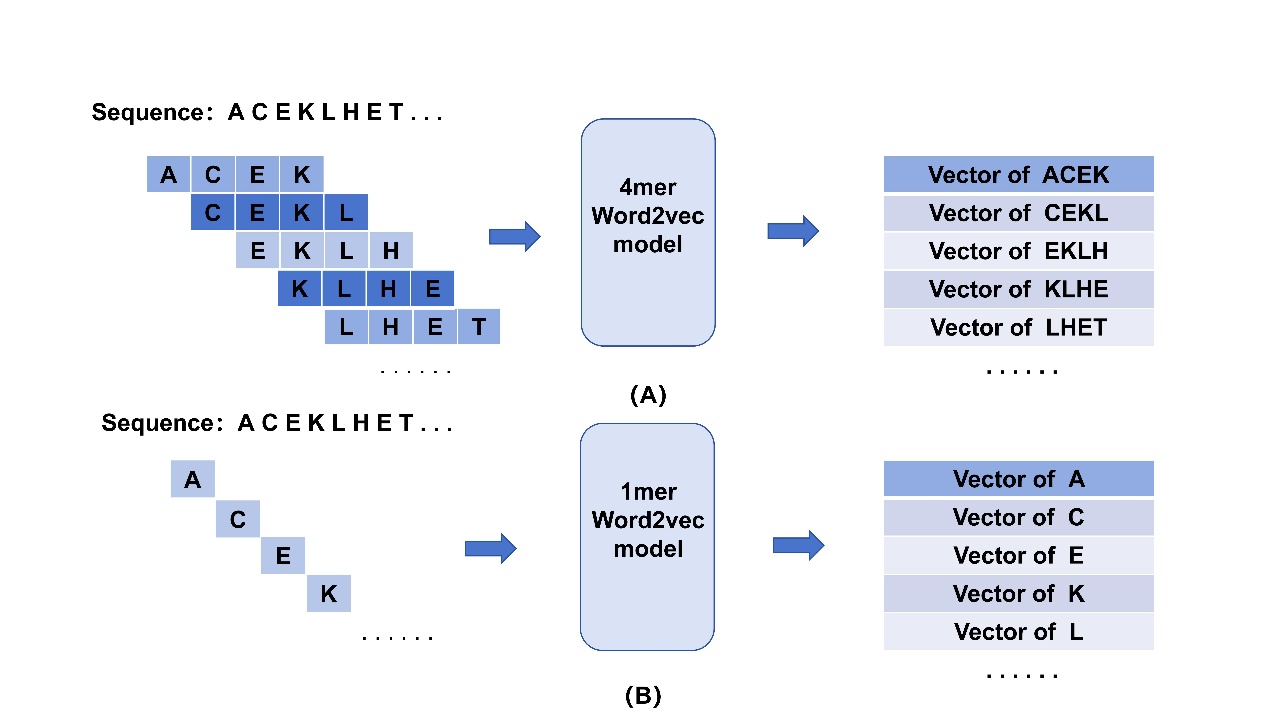


**Figure S2**. Word2Vec models trained based on words of 1mer (A) and 4mers (B)


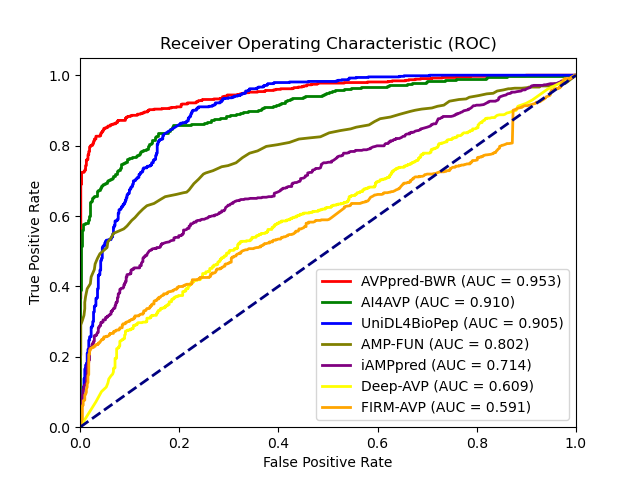


**Figure S3.** ROC curves of different methods on the independent test set.


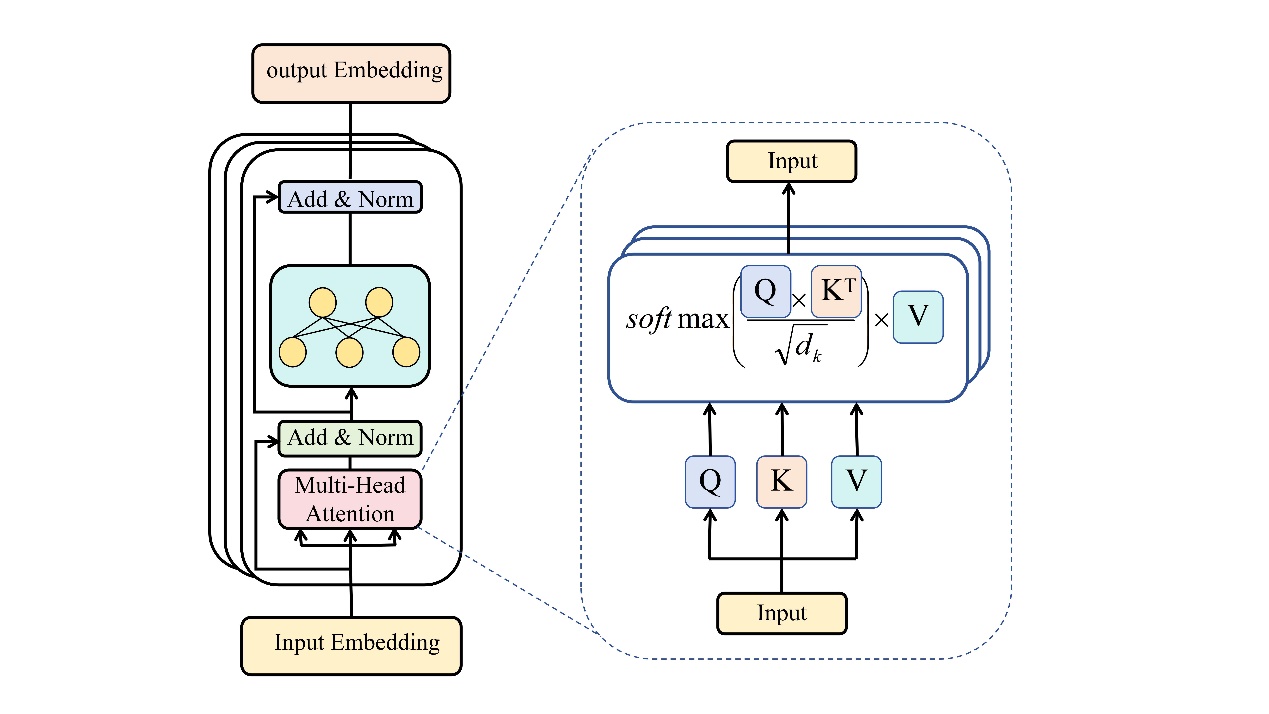


**Figure S4.** Diagram for the Encoder of Transformer.
